# In-hospital costs of malignant brain tumor surgery: A systematic review and quality assessment

**DOI:** 10.1093/nop/npaf121

**Published:** 2026-01-22

**Authors:** Floor R Pijl, Maik L Landers, Tanvi Kamra, Melissa Kerkhof, Maaike J Vos, Jaap D Zindler, Thijs van Der Vaart, Mar Rodríguez-Girondo, Rob J A Nabuurs, Marike L D Broekman, Wouter A Moojen, Wilco C Peul, Jeroen T J M van Dijck, Rishi D S Nandoe Tewarie

**Affiliations:** Department of Neurosurgery,University Neurosurgical Center Holland, Haaglanden Medical Center, The Hague, The Netherlands; Department of Neurosurgery,University Neurosurgical Center Holland, Haaglanden Medical Center, The Hague, The Netherlands; Department of Neurosurgery,University Neurosurgical Center Holland, Haaglanden Medical Center, The Hague, The Netherlands; Department of Neurology, Haaglanden Medical Center, The Hague, The Netherlands; Department of Neurology, Haaglanden Medical Center, The Hague, The Netherlands; Department of Radiation Oncology, Haaglanden Medical Center, The Hague, The Netherlands (J.D.Z.); Department of Neurology, Haaglanden Medical Center, The Hague, The Netherlands; Department of Medical Statistics, Leiden University Medical Center, Leiden, The Netherlands (M.R.-G.); Department of Neurosurgery,University Neurosurgical Center Holland, Haaglanden Medical Center, The Hague, The Netherlands; Department of Neurosurgery,University Neurosurgical Center Holland, Haaglanden Medical Center, The Hague, The Netherlands; Department of Neurosurgery, University Neurosurgical Center Holland, Leiden University Medical Center, Leiden, The Netherlands; Department of Neurosurgery,University Neurosurgical Center Holland, Haaglanden Medical Center, The Hague, The Netherlands; Department of Neurosurgery, University Neurosurgical Center Holland, Leiden University Medical Center, Leiden, The Netherlands; Department of Neurosurgery,University Neurosurgical Center Holland, Haaglanden Medical Center, The Hague, The Netherlands; Department of Neurosurgery, University Neurosurgical Center Holland, Leiden University Medical Center, Leiden, The Netherlands; Department of Neurosurgery,University Neurosurgical Center Holland, Haaglanden Medical Center, The Hague, The Netherlands; Department of Neurosurgery, University Neurosurgical Center Holland, Leiden University Medical Center, Leiden, The Netherlands; Department of Neurosurgery,University Neurosurgical Center Holland, Haaglanden Medical Center, The Hague, The Netherlands; Department of Neurosurgery, University Neurosurgical Center Holland, Leiden University Medical Center, Leiden, The Netherlands

**Keywords:** brain tumor surgery, cost drivers, in-hospital costs, intracranial malignancies, systematic review

## Abstract

The increasing financial burden of therapies for brain tumor patients necessitates strategies that preserve equitable access and optimize resource allocation. The aim of this systematic review was to evaluate the in-hospital costs for malignant brain tumor surgery. A systematic review was carried out according to PRISMA guidelines on January 10, 2025, using the following databases: PubMed, MEDLINE, Embase, Web of Science, Cochrane Library, CENTRAL, CINAHL, PsycINFO, and Academic Search Premier. Articles published after January 1, 2000, describing perioperative hospital costs for malignant brain tumor surgery patients were included. Study quality assessment was performed based on the 2022 CHEERS statement. Nine studies were included, encompassing a total of 140 813 patients. Total in-hospital costs were highly variable and ranged between $2382.47 and $30 836.98 per patient. Mean length of stay (LOS) was 5.24 days, ranging from 3 to 7.12 days. LOS and in-hospital costs were positively correlated. Study quality was low due to missing data on health economic analysis plans, and lack of reporting of uncertainty in the findings. In-hospital costs for malignant brain tumors are high, rising over time, and were significantly impacted by LOS. Study outcomes showed high variability, poor methodological quality due to inconsistent methods and inadequate cost data. Future research needs standardized, comprehensive cost analyses to support informed healthcare policy and resource allocation decisions.

Key Points

- Brain tumor surgery costs are high globally and show a rising trend.
- Length of stay is a key driver regarding inpatient expenses.
- Standardized costs analyses are necessary for economic evaluation of malignant brain tumor surgery.

Importance of the Study

Malignant brain tumors represent one of the most challenging and costly conditions in oncology, combining high treatment expenses with overall low survival. As a primary therapeutic modality, surgical management constitutes as a significant cost driver due to the intense resource utilization, ever-advancing technology, and intensive peri- and postoperative care. This raises critical questions regarding cost-effectiveness and sustainability of current treatment strategies. With healthcare costs escalating globally, driven by aging populations, increased incidence of intracranial malignancies and the development of advanced therapies, understanding the economic impact of malignant brain tumor surgery is crucial. Cost analyses are essential for accurate policy decisions, optimal resource allocation, and to support value-based care models. Moreover, these analyses can identify the missing data necessary to critically evaluate treatment and healthcare costs. This systematic review addresses this knowledge gap by summarizing evidence on in-hospital costs in brain tumor surgery, cost drivers, and methodological quality of current studies.

As costs rise globally, access to healthcare becomes increasingly threatened.^1^^,^^2^ Direct cost estimates vary substantially between institutions and countries, making a true assessment of costs difficult.^3^ Brain cancer carries a significant economic impact, estimated at €37.8 billion in Europe in 2019, with annual per patient cost of €189 299.^4^ Brain and central nervous system (CNS) tumors are ranked as the seventh most costly cancer type, despite having a considerably lower incidence rate.^5^^,^^6^ For example, their associated economic burden is comparable to that of stomach cancer, even though brain tumors have a lower in worldwide incidence globally. These costs are mainly driven by intensive initial treatments, significant utilization of inpatient care, pharmacy-dispensed drugs, outpatient visits, and emergency services.^3^^,^^7^ Moreover, rising patient numbers have significantly contributed to increased cost of brain cancer care in Europe over the last decade.^8^^,^^9^ With an aging population and adoption of novel costly therapies, potentially improving survivorship, expenditures are expected to rise further.^10-12^

Despite high treatment costs for primary malignant brain and CNS tumors, outcomes remain poor. The overall 5-year survival rate refrains from improving and averages 36% overall and for glioblastomas just 7%.^13-15^ This underscores the need to weigh treatment costs against quality of life during limited survival.^16^ Quality-adjusted life years (QALYs) offer a framework for evaluating cost-effectiveness, with current therapies ranging massively from $8325 to $458 261 per QALY.^17^^,^^18^ With QALY thresholds ranging from $50 000 to $150 000, many of these therapeutic options remain points of discussion. These expenditures intensify upward cost trends and highlight the urgency of evaluating future therapeutic options.^19^ However, existing literature on cost-effectiveness is limited, therefore, not sufficient to support a systematic review.

Understanding in-hospital costs of brain tumor treatment and their drivers is essential for healthcare sustainability.^20^ Post-COVID-19, health expenditures increased significantly and remain above pre-pandemic level.^21^^,^^22^ These growing expenses demand optimized resource allocation. Making cost data available to policymakers, clinicians, and politicians is crucial for informed decision-making while maintaining quality of care.^16^^,^^23-25^ To our knowledge, there are no recent attempts to estimate the costs of malignant brain tumor surgery. This systematic review therefore examines reported in-hospital costs of treating malignant brain tumors, assessing outcomes as well as methodological quality.

## Methods

This systematic review was performed in accordance with the Preferred Reporting Items for Systematic Reviews and Meta-Analyses (PRISMA) guidelines (Appendix 1).^26^ The study protocol was registered in the PROSPERO International Prospective Register of Systematic Reviews under CRD420250642443.

### Literature Search

A literature search was conducted on January 10, 2025, using: PubMed, MEDLINE, Embase, Web of Science, Cochrane Library, CENTRAL, CINAHL, PsycINFO, and Academic Search Premier. The final strategy was developed with a trained librarian. No filters or limits were applied. Details are provided in Appendix 2. Reference lists of included articles were also screened for additional studies

### Inclusion and Exclusion Criteria

Studies were included if they reported in-hospital costs for ≥10 patients with intracranial malignancies, defined as either primary malignant brain tumors or cerebral metastases. Studies on WHO CNS grade 1 tumors brain were excluded. In-hospital costs were defined as all inpatient and outpatient costs from the day of admission until 30 days after surgery and had to be distinguishable from other reported costs. Studies reporting only inpatient or outpatient costs within this period were also included. Exclusion criteria were: publication before January 1, 2000; inclusion of patients <18 years; unpublished or inaccessible full texts; non-English/Dutch language; and non-original data such as reviews, editorials, commentaries, textbook, and conference meetings/abstracts.

### Article Selection

Duplications were first removed. Four reviewers (F.R.P., M.L.L., J.T.J.M.v.D., R.D.S.N.T.), separately independently screened titles and abstracts for eligibility. Full-text screening was conducted by two reviewers according to the inclusion criteria. Disagreements were resolved by consensus, with a senior reviewer consulted when necessary.

### Data Extraction

Data extraction was performed independently by two researchers using a pre-determined data extraction document. Given the heterogeneity of studies, both conducted a manual synthesis after which data were compared and finalized. The following data were collected for each article: study design, country, year of publication, aim, cohort size, malignancy type, outcomes, study currency, cost data sources, methodology, healthcare consumption, cost outcomes, and Gross Domestic Product (GDP). Final costs were expressed as a percentage of national GDP.

### Quality Assessment

The evaluation of in-hospital cost studies for intracranial malignancies was based on the CHEERS 2022 statement, an updated version of the 2013 guideline for standardizing health economic evaluations.^27^ No modifications were made to ensure the integrity of the quality assessment. The checklist comprised twenty-eight items covering study details, population, cost data, and methodology (Appendix 3).

Two reviewers independently conducted the assessment. Disagreements were resolved by discussion until consensus. Items were scored as yes (1), no (0), and not applicable (N/A). Final quality scores were calculated as a percentage of the maximum attainable score, with additional results reported per question and category. N/A items were excluded from calculations.

### Outcomes

Reported data were presented descriptively. Diagnosed malignancies were reported as specified in studies or, if unclear, as not further specified. Currency and reference year were recorded and if missing, the last year of patient inclusion was used as reference year. To improve comparability, costs were expressed relative to national GDP per capita, standardized to the study’s currency year.^28^ All values were converted to 2015 US dollars using the CCEMG–EPPI-Centre Cost Converter.^29^ This tool applies employs International Monetary Fund GDP deflators and Purchasing Power Parities rates.^30^ If no reference year was reported, the last inclusion year was used. Mean and median values were calculated using IBM SPSS Statistics for Windows, Version 29.0, which was also used for graph generation.

## Results

### Literature Search and Study Selection

**Figure 1.**
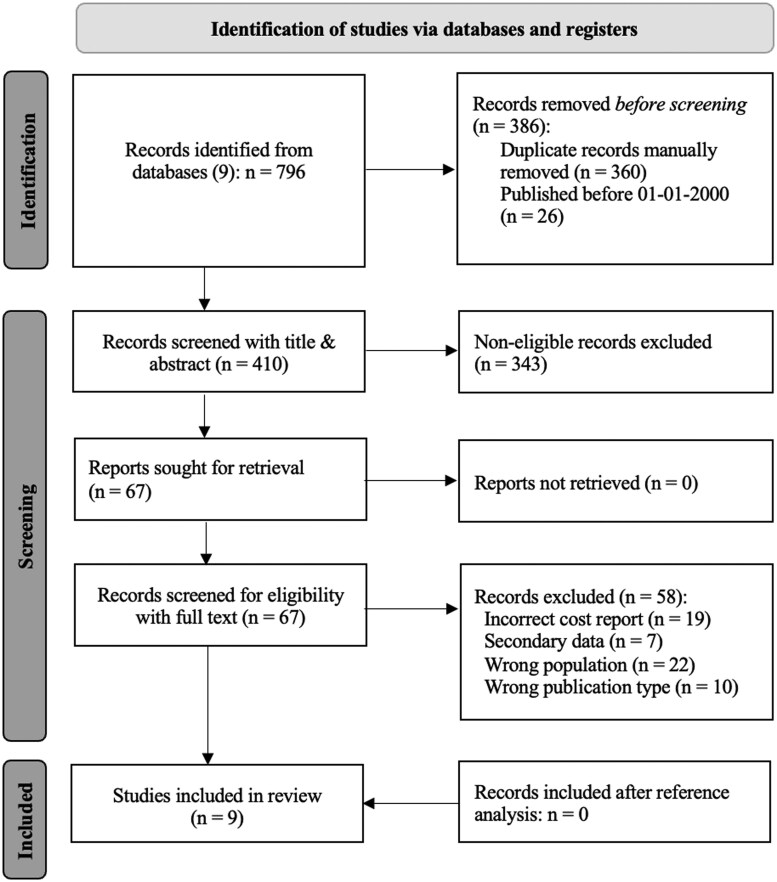
Flow chart of study selection process. The study selection flow chart according to the PRISMA 2020 flow diagram for new systematic reviews.

The search identified 796 studies. After removal of 360 duplicates and 26 studies published before January 10th, 2000, 410 records remained. Title and abstract screening excluded 343 studies, leaving 67 for full-text assessment. Of these, nine studies met all inclusion criteria and were included in this review (Figure 1).^31-39^

### Study Characteristics

Study characteristics are summarized in Table 1. Four studies were published between 2000 and 2019, and five between 2020 and 2022. All patients were treated before the COVID-19 pandemic. Cohort sizes ranged from 22 to 55 203 patients,^32^^,^^39^ with a total of 140 813 patients. Most studies were conducted in high-income countries; one single-center study was from Brazil.^34^ Three studies were multicenter, all American database studies.^35^^,^^38^^,^^39^ Eight of nine studies included cost research in their objectives. No randomized controlled trials or experimental treatments were included. One study was prospective, and two had mixed retrospective-prospective designs.^34^^,^^36^^,^^37^ The remaining studies (67%) had a retrospective design.

**Table 1.** Study characteristics

Study no.	Study info^a^	Study design	Research aim	Patients (N)	Diagnosed malignancy	Outcomes	Cost data source	Cost calculation	Included costs	Currency (Y)	GDP per capita	Results^b^ ($ 2015) (% of GDP per capita)
1	Manuel et al^31^ 2022 2015–2019 USA Single-center	Retrospective cohort study	Evaluate the influence of LEP on experiences, inefﬁciencies and costs in brain tumor craniotomies	Total 2232 150 LEP 2082 EP	Brain tumors (not further specified)	LOS, direct costs, discharge disposition, 30-day readmission rate	Electronic health data	NS, most likely obtained directly from health data	Direct costs of hospitalization	US$ (NS)	$61 048	Total: $21 974.88 (36%) (IQR: $17 857.63 -$28 464.68) LEP: $25 507.14 (42%) (IQR: $19 684.63 - $33 266.36) EP: $21 827.98 (36%) (IQR: $17 811.58 - $27 960.79)
2	Martino et al^32^ 2012 2009–2011 Spain Single-center	Retrospective matched-pair study	Determine cost-effectiveness of awake glioma surgery for tumors in eloquent areas	Total 22 11 awake and IES 11 GA and no IES	Isolated WHO grade II gliomas (astrocytoma, oligodendroglioma or mixed glioma) without multicentric or multifocal extension in an eloquent area	Utility (QALY’s), direct costs, indirect costs	Spanish research center (SOIKOS), Spanish Vademecum (drugs)	Hospital database, number of resources multiplied by unit costs for each cost component	Direct medical costs, hospital costs, surgery costs, costs of drugs	US$ (2011)	$25 510.60	Mean hospitalization costs: IES: $9261.43 (36%) (range $8247.74 – $11 964.60) No IES: $8923.53 (35%) (range $7410.30 – $13 022.96) Mean surgery costs: IES: $9707.87 (38%) (range $6142.01 – $14 470.74) No IES: $3488.68 (14%) ($2626.51 – $4450.41) Calculated combined mean hospital and surgery costs: IES: $9484.65 (37%) (range $7194.88 - $13.217.67) No IES: $6206.11 (24%) (range $5018.41 - $8736.69)
3	Nassiri et al^33^ 2018 2014–2015 Canada Single-center	Retrospective chart review	Compare costs associated with inpatient vs outpatient awake craniotomy	Total 50 29 outpatient 21 inpatient	Supratentorial tumors (not further specified)	Direct and indirect costs (physician fees excluded)	University Health Network Case Costing Department and Ontario Case Costing Initiative	Standardized case-costing analysis	Direct costs (patient departments) and indirect costs (non-patient departments)	CA$ (NS)	$43 594.20	Inpatient craniotomy: $8800.83 (20%) Outpatient craniotomy: $4332.23 (10%)
4	Neville et al^34^ Single-center	Partial retrospective cohort study	Evaluate the effect of the DAHD in brain tumor resection patients	Total 66 32 pre-DAHD 34 DAHD	Craniotomy for brain tumor resection (not further specified)	LOS, 30-day complication rate, 30-day hospital costs	Not specified	Absorption costing method and micro-costing method	30-day hospital costs	US$ (2018)	$8722.30	Total: $2811.33 (32%) ($1936.53–$4387.31) Pre-DAHD: $3380.62 (39%) ($2172.78–$4663.41) DAHD: $2382.47 (27%) ($1704.07–$3920.49)
5	Tang et al^35^ 2020 2006 and 2009 USAMulticenter	Retrospective database study	Evaluate if interhospital competition alters inpatient charges and costs in cranial neurosurgery	65 168 tumor related craniotomies	Tumor patients undergoing cranial neurosurgery (not further specified)	Inpatient charges, inpatient costs, LOS	NIS datasets, CMS (cost-to-charge ratios)	Multiplying charges with hospital-specific cost-to-charge ratios	Inpatient costs	US$ (2009)	$51 863.60	2006: $26 374.37 (51%) SEM ± $264.42 2009: $27 040.91 (52%) SEM ± $244.58
6	ter Laan et al^36^ 2020 2016–2018 NL Single-center	Partial retrospective cohort study	Provide a quieter postoperative environment, reduce ICU burden and evaluate possible cost reduction	Total 216 Pre-protocol (A) 107 Post-protocol (B) = 109	Elective craniotomy for supratentorial tumors (not further specified)	30-day complication rate, LOS, patient satisfaction, hospital costs	Hospital billing department (third-party payer perspective)	NS, most likely obtained directly from billing department	Mean total costs per admittance	€ (NS)	$48 463	Cohort A: $16 161.56 (33%) Cohort B: $13 841.91 (29%)
7	ter Laan et al^37^	Prospective cohort study	Comparing supratentorial tumor craniotomies after introduction of a “no ICU, unless policy”	Total 365 Pre-protocol (A) 107 Post-protocol (B) 258	Elective open craniotomy for supratentorial tumors (not further specified)	30-day complication rate, LOS, patient satisfaction, hospital costs	Hospital billing department (adhering to a third-party payer perspective)	NS, most likely obtained directly from billing department	Mean total costs per admittance (EMM)	€ (NS)	$49 254	Cohort A: $15 224.75 (31%) Cohort B: $13 274.25 (27%)
8	Zhang et al^38^ 2020 2007–2016 USA Multicenter	Retrospective longitudinal cohort study	Assess operative microscope use in supratentorial resections	Total 17491 12 058 glioma patients, matched 4993 5433 metastasis patients, matched 1985	Patients with glioma or brain metastases who underwent supratentorial resection with or without use of operative microscope	Intraoperative microscope use, 30-day readmissions, time to first readmission, 30-day complication rate and total 30-day healthcare cost	Truven Health MarketScan Commercial Claims and Encounters, Medicare Supplemental and Coordination of Benefits databases	NS, most likely obtained directly from databases	Mean 30-day postoperative cost (total eligible charges incurred within 30 days of surgery)	US$ (NS)	$57 430.80	Gliomas: Microscope: $7523.12 (13%) (SEM $552.56) No microscope: $6844.52 (12%) (SEM $1863.82) Metastases: Microscope: $23 485.97 (41%) (SEM $832.66) No microscope: $22 586.98 (39%) (SEM $3433.10)
9	Zygourakis et al^39^ 2017 2001–2013 and 2012–2015 USA Multicenter	Retrospective database study	Examine patient and hospital factors that influence cost variation in tumor craniotomies	Total 55 203 NIS database 41 483 patients Vizient CDM/RM database 13 720 patients	Primary surgery for supratentorial brain tumors	Mean LOS, mean ICU days and weighted mean hospitalization costs	NIS database, Vizient CDM/RM database	NIS: total hospital charges, all-payer inpatient cost-to-charge ratio’s Vizient CDM/RM: adjusted costs, area wage indices	Total direct hospital costs	US$ (2013)	$54 728.90	NIS (2013): $30 836.98 (56%) (SE $665.69) Vizient CDM/RM (2013): $29 554.98 (54%) (SE $7313.38)

Abbreviations: CMS: Centers for Medicare and Medicaid Services; DAHD: daily algorithm for hospital discharge; EMM: estimated marginal means; GA: general anaesthesia; HHI: Herfindahl-Hirschman Index; ICU: intensive care unit; IES: intraoperative electrical stimulation; LEP: limited English proficiency; LOS: length of stay; MCU: medium care unit; MU: microscope use, NL: the Netherlands; NIS: National Inpatient Sample; NMU: no microscope use; NS: not specified; EP: English proficiency; QALY’s: quality adjusted life years; SE: standard error; SEM: standard error of the mean; USA: United States of America.

a Name of the first author [reference no.], publication year, cohort inclusion years/period—study country—single- or multicenter.

b GDP per capita of reference year of currency, converted to 2015 US dollars.

Intracranial malignancies were most commonly defined as “brain tumors” or “supratentorial brain tumors” (78%).^32^^,^^38^ Follow-up for cost data was stated as 30 days in three studies,^31^^,^^34^^,^^38^ the others reported all inpatient costs for primary tumor resection.^32^^,^^33^^,^^35-37^^, ^^39^ Some studies lacked complete data: five did not report the reference year of currency,^31^^,^^33^^,^^36-38^ four did not clearly define the cost calculation method (44%),^31^^,^^36-38^ and one did not state the source of cost data.^34^ For Zygourakis et al, only 2013 cost data were included in Table 1, as this year was reported in both national databases.^39^ In Martino et al., mean hospital and surgery costs were combined to estimate inpatient costs with a corresponding range.^32^

### In-Hospital Cost Data

**Figure 2.**
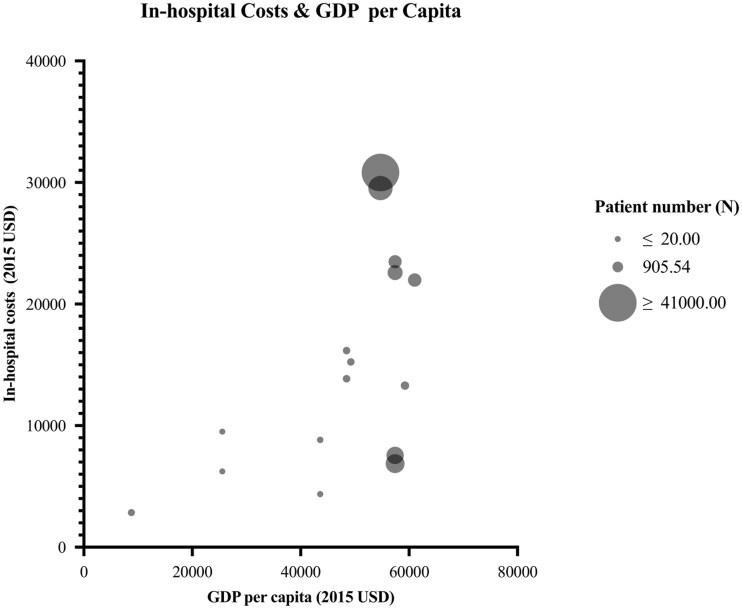
In-hospital costs versus GDP per Capita. Circle size indicates the sample size; circle colours represent the income category of the study country (high-income of low-income country). GDP per capita and in-hospital costs were converted to 2015 USD. One study was not included in this graph, since no separate sample size was provided for both study cohorts.

Reported in-hospital costs per patient were highly variable in the included studies. In-hospital costs per study country included in this review are summarized in Figure 2. The lowest costs ranged from $2382.47 to $6844.52 and were reported in studies performed in a middle-income country analyzing early hospital discharge after craniotomy and one comparing inpatient and outpatient craniotomies.^33^^,^^34^^,^^40^ The highest in-hospital costs reported ranged from $26 374.37 to $30 836.98 and were seen in two American database studies.^35^^,^^39^ The in-hospital costs as a percentage of the GDP per capita differed largely in all studies, ranging between 10% and 56%. Mean and median percentages were calculated as 34% and 35%, respectively. The mean and median for high-income countries (eight out of nine studies) did not significantly differ from the overall values or the singular study performed in a middle-income country. Percentages were the highest in a large American database study.^39^

**Figure 3.**
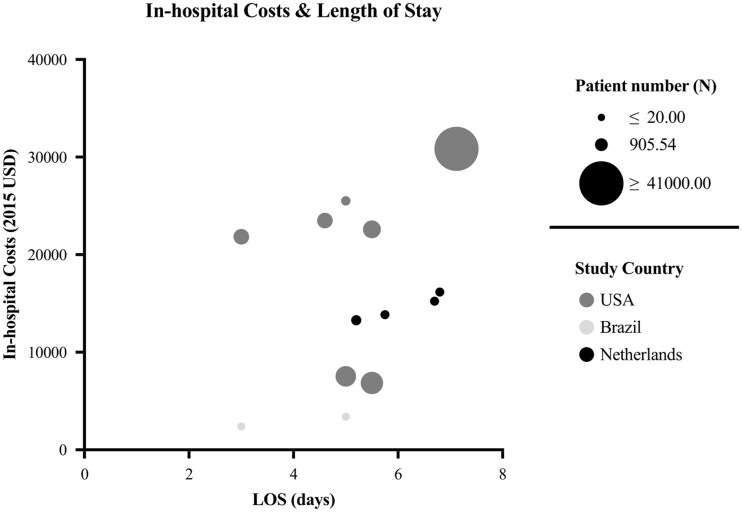
In-hospital costs versus LOS. All cohorts reporting LOS were included. Circle size reflects cohort size; circle color indicates the study country.

Several studies reported the length of stay (LOS) after tumor-related craniotomy.^31^^,^^34-39^ The mean LOS across all cohorts in these studies was 5.24 days and ranged from 3 to 7.12 days. Four studies reported the LOS to have a significant impact on the in-hospital costs for craniotomy.^34^^,^^36^^,^^37^^,^^39^ Three studies investigated postoperative ICU admissions and discharge protocols.^34^^,^^36^^,^^37^ One study reported on admission to the intensive care unit (ICU) LOS, which was 0.47 and 0.23 days in two cohorts comparing standard ICU admission to a “No-ICU unless” policy. The same study also reported on admission to medium care unit (MCU) LOS, with an average of 0.53 days in both cohorts.^36^ A reduction in ICU admission time did not lead to a longer stay at the neurosurgical ward.^36^^,^^37^ Consequently, shorter ICU stays accounted for 75% of the $1922.67 reduction per patient. All studies reported reduced 30-day in-hospital costs with lower admission to the care units. Two studies demonstrated that a “No-ICU unless” policy lowered costs by 13% (*P* < .0001) without compromising safety.^36^^,^^37^ Four studies^34^^,^^36-38^ reported on complication rates, where two articles showed fewer complications with selective postoperative ICU admissions.^36^^,^^37^ Another study reported a significant increase in in-hospital costs due to an extended stay in the neurosurgical ward ($1539.92 vs $874.80, *P* = .009), with similar ICU LOS.^32^ Zygourakis et al determined that a longer LOS was associated with significantly higher tumor craniotomy costs.^39^ A discharge algorithm shortened overall LOS and reduced hospitalization costs (*P* = .043), although 30-day costs were not significantly different. ($2811 vs $2382, *P* = .112).^34^ Outpatient craniotomies incurred substantially lower costs than inpatient procedures (*P* < .001), especially in unit/bed costs (6-fold decrease, $3423 vs $626, *P* < .001), while operating room, anesthesia, and laboratory costs were similar.^33^ Readmission rates (30-day) (8.6% and 19.9%) showed no significant change. As displayed in Figure 3, total LOS and in-hospital costs exhibited a positive correlation.

Only one study reported other cost drivers, such as limited English language proficiency (LEP, reported as patient with a non-English primary language and preference for interpreter services) ($25 507 vs $21 975, *P* < .001),^29^ male gender, private insurance, higher mortality risk, severity of illness, nonelective admission, and wage index.^37^ Increased costs also stemmed from pharmacy, imaging, food and support services.^31^ Different tumor pathology strongly influenced costs: metastasis cohorts ($23 486 and $22 587) exceeded glioma cohorts (ranging from $6206 to $9485).^36^

Finally, in-hospital costs have shown an upward trend over time. Craniotomy costs increased from $23 201 in 2001 to $29 971 in 2013, a 13% rise.^37^ Similarly, a separate analysis showed an average increase in inpatient costs of $666.54 between 2006 and 2009, although this change did not reach statistical significance (*P* = .064). In contrast, inpatient charges rose significantly during the same period (*P* < .001), a trend that was positively associated with increased hospital competition (attracting patients by adjustment of multiple factors like price or quality of services), as measured by the Herfindahl-Hirschman Index.^33^ More recent data on cost trends were not reported in the included articles.

### Quality Assessment of Study Methodology

**Figure 4.**
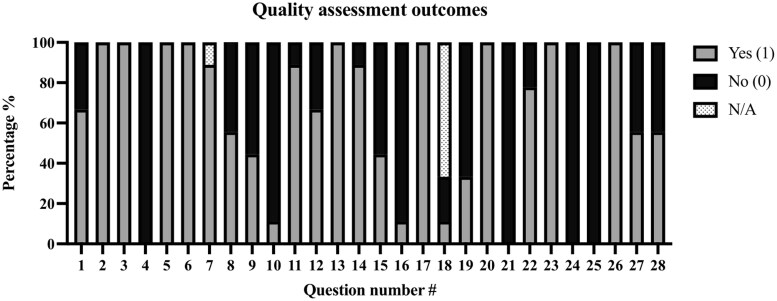
Quality assessment outcomes. Scores of included articles using the CHEERS 2022 statement. Scores are categorized as: Yes (1 point, gray) indicates the item was completely reported; No (0 points, black) indicates the item was not reported at all or according to the criteria; N/A (dotted) indicates the item was not applicable.

Results of the CHEERS 2022 quality assessment are shown in Table 2 and Figure 4. Study quality ranged from 50% to 70%, with an average score of 62%, indicating low-to-medium methodological quality. Six of the nine studies (67%) clearly identified as economic evaluation in the title.^33-36^^,^^38^^,^^39^ None reported a health economic analysis plan; one study used modeling,^39^ and discounted rates were applied in only one study.^32^ Three studies identified subgroups, with one performing a subgroup analysis.^35^^,^^36^^,^^38^ Five studies reported the study perspective and time horizon, but did not justify them, resulting in zero point for these items.^31-33^^,^^38^^,^^39^ All but one study described how costs were valued.^32^^,^^39^ Four reported resource quantities and currency details.^32^^,^^34^^,^^35^^,^^39^ All studies reported mean cost outcomes, and seven (78%) addressed uncertainty in analytic inputs, though none evaluated its impact on findings.^31^^,^^32^^,^^34-36^^,^^38^^,^^39^ Eight studies (89%) disclosed conflicts of interest^31^^,^^32^^,^^34-39^ and five reported funding sources.^31^^,^^32^^,^^34^^,^^36^^,^^37^

**Table 2.** Results of CHEERS 2022 quality assessment

Quality assessment checklist		Reference no.		1	2	3	4	5	6	7	8	9	Score (%)
Item	No.	Question											
Title	1	Is the study identified as an economic evaluation and are the compared interventions specified?		0	0	1	1	1	1	0	1	1	67
	Subtotal score (N/1)			0	0	1	1	1	1	0	1	1	67
Abstract	2		Is a structured summary provided which highlights context, key methods, results and alternative analyses?	1	1	1	1	1	1	1	1	1	100
	Subtotal score (N/1)			1	1	1	1	1	1	1	1	1	100
Introduction	3		Is context for the study, the study question and its practical relevance for decision making in policy or practice given?	1	1	1	1	1	1	1	1	1	100
	Subtotal score (N/1)			1	1	1	1	1	1	1	1	1	100
Methods	4		Is it indicated whether a health economic analysis plan was developed and where this is available?	0	0	0	0	0	0	0	0	0	0
	5		Are characteristics of the study population (eg age, demographics) described?	1	1	1	1	1	1	1	1	1	100
	6		Is relevant contextual information that may influence findings provided?	1	1	1	1	1	1	1	1	1	100
	7		Are the interventions or strategies being compared described and is explained why they were chosen?	1	1	1	1	1	1	1	1	NA	100
	8		Is the study perspective(s) and the reason for choosing stated?	0	0	1	0	1	1	1	0	0	44
	9		Is the time horizon for the study and why this was appropriate stated?	0	0	0	1	1	1	1	0	0	44
	10		Are discount rate(s) and the reason for choosing stated?	0	1	0	0	0	0	0	0	0	11
	11		Are the outcomes were used as the measure(s) of benefit(s) and harm(s) described?	1	1	1	1	0	1	1	1	1	89
	12		Is described how outcomes used to capture benefit(s) and harm(s) were measured?	1	1	0	1	0	1	1	1	0	67
	13		Are the population and methods used to measure and value outcomes described?	1	1	1	1	1	1	1	1	1	100
	14		Is described how costs were valued?	0	1	1	1	1	1	1	1	1	89
	15		Are the dates of the estimated resource quantities and unit costs, plus the currency and year of conversion reported?	0	1	0	1	1	0	0	0	1	44
	16		Is modeling used? If yes, is it described in detail and the reason why explained? (report if the model is publicly available and where it can be accessed)	0	0	0	0	0	0	0	0	1	11
	17		Are any methods for analyzing or statistically transforming data, any extrapolation methods, and approaches for validating any model used described?	1	1	1	1	1	1	1	1	1	100
	18		Are any methods used for estimating how the results of the study vary for sub-groups described?	NA	NA	NA	NA	0	0	NA	1	NA	33
	19		Is described how impacts are distributed across different individuals or adjustments made to reflect priority populations?	1	0	0	0	1	0	0	1	0	33
	20		Are methods to characterize any sources of uncertainty in the analysis described?	1	1	1	1	1	1	1	1	1	100
	21		Are any approaches to engage patients or service recipients, the general public, communities, or stakeholders (eg clinicians or payers) in the design of the study described?	0	0	0	0	0	0	0	0	0	0
	Subtotal score (N/18)			9	11	9	11	11	11	11	11	9	60
Results	22		Are all analytic inputs (eg values, ranges, references) including uncertainty or distributional assumptions reported?	1	1	0	1	1	1	0	1	1	78
	23		Are the mean values for the main categories of costs and outcomes of interest reported and summarized in the most appropriate overall measure?	1	1	1	1	1	1	1	1	1	100
	24		Is described how uncertainty about analytic judgments, inputs, or projections can affect findings? (report the effect of choice of discount rate and time horizon, if applicable)	0	0	0	0	0	0	0	0	0	0
	25		Are any difference patient/service recipient, general public, community, or stakeholder involvement made to the approach or findings of the study reported?	0	0	0	0	0	0	0	0	0	0
	Subtotal score (N/4)			2	2	1	2	2	2	1	2	2	44
Discussion	26		Are key findings, limitations, ethical or equity considerations not captured, and how these could impact patients, policy, or practice reported?	1	1	1	1	1	1	1	1	1	100
	Subtotal score (N/1)			1	1	1	1	1	1	1	1	1	100
Other relevant information	27		Is described how the study was funded and any role of the funder in the identification, design, conduct, and reporting of the analysis?	1	1	0	1	0	1	1	0	0	56
	28		Is reported if authors have conflicts of interest according to journal or International Committee of Medical Journal Editors requirements?	1	1	0	1	1	1	1	1	1	89
	Subtotal score (N/2)			2	2	0	2	1	2	2	1	1	72
Total score (in %, maximum score 28)				16p 50%	18p 67%	14p 52%	20p 70%	18p 64%	19p 68%	17p 63%	18p 64%	16p 62%	

## Discussion

This systematic review illustrates that overall in-hospital costs of malignant brain tumor patients are generally high and take up a substantial percentage of the GDP per capita in all included countries. These expenditures were comparable to other common cancer types. Costs were positively associated with total LOS, although studies demonstrated a wide variability. Key cost drivers identified were LOS and ICU LOS. Clinical and methodological heterogeneity was present in our review, most likely contributing to the observed variability in in-hospital costs across all studies.

When comparing the results of our review on in-hospital costs to the existing literature, reviews assessing total costs of various brain disorders show a similar trend of a high financial burden. A large review published in 2025 reported direct healthcare costs of €15 258 per patient and average total costs per patient per year of €189 229 in Europe. This is in line with previous publications on known heterogeneity in pathology, differences between institutions and countries and cost criteria.^3^^,^^4^ When comparing to other neurosurgical subspecialties, a study showed that tumor surgery had higher costs compared to vascular and functional neurosurgery, but lower than neurotrauma and CSF shunt craniotomies.^35^ Comparing our cost data to other cancer types illustrates a similar trend of overall high, but varying expenditures. Lung, colorectal, and breast cancer are reported to have inpatient costs ranging from $22 360 to $37 014, illustrating a slightly higher, but generally similar trend.^41^

In accordance with literature, LOS was reported to significantly increase in-hospital costs.^34^^,^^39^^,^^42-45^ Earlier discharge resulted in decreased ward costs, as well as pharmacy, imaging, and food and support services spending.^34^^,^^39^ Furthermore, a reduction in ICU stay was reported to significantly lower costs. Consistent with these findings, outpatient craniotomy showed a decreasing trend of costs.^33^^,^^34^^,^^44^ LOS is also likely to play a role in the significant increase of in-hospital costs of LEP patients and non-elective admissions.^31^ Even though no literature is available regarding malignant tumors, research on meningioma patients shows that patient-related socioeconomic factors may correlate with LOS, costs and healthcare consumption.^46^^,^^47^ Elective brain tumor resections are associated with significantly lower total hospital charges and costs because of decreased LOS and mortality risk.^48^^,^^49^

Differences in pathology-specific costs may have attributed to greater healthcare resource utilization, but may vary substantially.^50^^,^^51^ Based on our limited data in a cohort of metastasis patients compared to those with gliomas, higher mean costs were exhibited in metastasis patients, after adjusting for multiple confounders. This underlined the effect of overall patient health on 30-day expenditures. In general, metastatic patients have more comorbidities and demonstrated a higher mean age, possibly influencing these increased expenditures. Moreover, patients with brain metastases have often received multimodal treatment, resulting in even higher costs depending on the primary tumor.^52^^,^^53^

Two large database studies from the United States reported the highest in-hospital costs.^35^^,^^39^ Average American healthcare spending is about $4000 higher than other high-income countries, due to increased spending on professional services, especially prescription drugs.^54^^,^^55^ Moreover, literature reports significantly higher perioperative laboratory costs in the United States compared to the Netherlands.^56^ Both database studies evaluated costs by multiplying hospital charges by hospital-specific cost-to-charge ratios, which can cause overestimation of the total costs. Charges can differ from actual costs and can be tackled by assessing resource utilization based on the costs per unit.^57^ The lowest in-hospital costs were seen in 2 studies evaluating an intervention to decrease duration of admission, with one being performed in a middle-income country.^33^^,^^34^ Low- and middle-income countries have lower average spending on malignant brain tumors, as a result of lower resource costs but also poorer healthcare accessibility.^58^^,^^59^ Despite this, the average in-hospital costs as a percentage of the GDP per capita did not significantly differ between high-income countries compared to the single middle-income country study. Although the literature is limited and results can be debated, this suggests a comparable financial burden for patients in general.

The need for cost-analysis studies is becoming increasingly essential with the escalating pressure on healthcare expenses. The urge to minimize growth in healthcare-related costs has become vital due to the steep incline in expenditures during and after the COVID-19 pandemic.^21^ Even though none of our studies included a cohort of any pandemic or post-pandemic years, most studies observed a clear upward trend of in-hospital costs over the years.^35^^,^^39^ Available data regarding overall costs for brain tumor patients appears to be outdated in the light of the current post-pandemic healthcare environment which is substantially influenced by inflation and increasing social conflict. Consequently, with recent reports of overall healthcare costs continuing to increase in recent years, it is to be expected that in-hospital costs of malignant brain tumors have followed this trend^21^^,^^55^ upwards. With the significant role of in-hospital costs compared to the total costs of brain tumor healthcare, it raises the question how to optimally limit these increasing spendings. Long-term costs for society should be considered, which were outside the scope of this review. Indirect costs, defined as production loss due to disease, contribute substantially to long-term societal expenses and have also been identified as a main cost driver in CNS tumors.^60^^,^^61^ Due to the overall aggressive nature of intracranial malignancies, indirect costs can spike as a result of a rapid decline in patient health.^62^ Interventions to reduce indirect costs could therefore significantly contribute to cost reduction in malignant brain tumor patients. Moreover, the substantial societal burden combined with limited survival can spark ethical discussions on which treatment is fitting for each patient group. This reinforces the need to implement QALYs to optimally evaluate cost-effectiveness, which has not been reported in existing literature for malignant brain tumor surgery.

Variability was observed in multiple aspects of all articles and was most likely caused by the different study objectives. Although most studies included cost research in their research aim, other objectives differed substantially. Heterogeneity is a well-documented issue in healthcare economic analysis. Even though methods to perform accurate comparisons are described, data availability remains an obstacle.^63^ Decreased methodological quality compromises study quality and the value of data directly. Our quality assessment concluded that, although variable, overall study quality was low, and missing data were frequently observed. None of the studies were assessed to be of high quality. Health economic analysis plans, engagement of third parties, or influence of uncertainty on findings were never mentioned. Regarding cost data, discount rates, and reference years for resource quantities and currency were reported poorly. Missing reference years can substantially influence results when converting costs to a standardized currency and corresponding year to improve comparability. Poor documentation of cost data was likely due to most studies not being identified as primary cost studies but instead focusing on safety or efficiency outcomes such as complication rate, readmission rate, or recurrence. Moreover, due to the range of timeframes incorporated in this systematic review, variability can be amplified even further. This substantially decreases the possibility to extrapolate these results to healthcare institutions.

### Strengths and Limitations

This systematic review included a comprehensive literature search, including multiple databases, providing an accurate overview of existing literature. Inclusion criteria were clearly documented to decrease heterogeneity as much as possible. Cost data was displayed in a standardized currency and reference year to improve comparability and interpretation of results. This review was composed using the PRISMA 2020 guidelines, and quality assessment was performed using the CHEERS 2022 statement. By applying standardized tools, evaluation of included articles is optimally objectified and replicable, although we acknowledge that some subjectivity is inherent in the assignment of the quality scores. Our review also had some limitations. Although careful selection was performed, articles including benign brain tumors were possibly present in this review. Multiple studies marginally specified the included pathologies, often only reporting “supratentorial lesions” as an inclusion criterion. Moreover, the variety of included tumor pathologies resulted in a more heterogeneous cohort. Therefore, supplementary studies are necessary to determine tumor-specific in-hospital costs and financial burden. Taking this into consideration, average in-hospital costs for intracranial malignancies may be subject to overestimation or underestimation. Due to the exclusion criteria, indirect costs were not considered in this review. This enabled us to clearly evaluate 30-day costs in malignant brain tumor surgery. A long-term follow-up period would provide improved insights for cost-analysis studies as a result of a more complete, comprehensive view. Additionally, heterogeneity and missing data observed in cost-related outcomes, compromising comparability. This resulted in the inability to statistically determine mean or median in-hospital costs or perform a meta-analysis. To optimize evaluation of available data, we converted costs to a standardized currency (USD) and currency year (2015) and calculated the cost as a percentage of the GDP per capita. Also, the primary the CHEERS 2022 statement is not officially validated as a quality assessment tool. However, due to the focus of CHEERS 2022 on the grade of financial data, we opted to use it for this review. Moreover, because of our strict inclusion criteria, a limited number of studies met the standards, resulting in a relatively small cohort in this systematic review. This underlines the dire need for new research with adequate methodological quality and cost evaluation.

### Future Research

This systematic review overviews the influence of identified cost drivers on healthcare consumption. These cost drivers can be utilized to combat increasing healthcare costs in the future. Some studies have evaluated outpatient craniotomy as feasible and comparable to inpatient craniotomy regarding patient safety and complications.^64-68^ Remote patient monitoring could decrease LOS while demonstrating positive outcomes in patient safety.^63^ Future research has to further explore these possibilities. Moreover, standardized cost analyses are essential to guide sustainable decision-making. Future studies should minimize heterogeneity by applying consistent inclusion criteria, clearly defining reported costs and initiatives such as CHEERS 2022. Incorporating long-term and indirect costs will further capture the full economic burden.

## Conclusion

In-hospital costs for patients with intracranial malignancies are high and continue to rise. These costs are significantly impacted by LOS and contribute to healthcare spending relative to GDP. Study heterogeneity and frequent missing data limit comparability across existing literature or extrapolation to current healthcare systems. Future research should adopt standardized, comprehensive cost analyses to support informed decision-making and efficient resource allocation in neuro-oncological care.

## Data Availability

The datasets generated and analyzed during the current study are available from the corresponding author on reasonable request.

## References

[1] Rakshit S , Amin K, Cox C. How does cost affect access to healthcare. *Peterson-KFF Health System Tracker*; 2024. https://www.healthsystemtracker.org/chart-collection/cost-affect-access-care/. Accessed September 6, 2025.

[2] World Health Organization. World bank and WHO: half the world lacks access to essential health services, 100 million still pushed into extreme poverty because of health expenses. Press Release. 2017;13.

[3] Goel NJ , Bird CE, Hicks WH, Abdullah KG. Economic implications of the modern treatment paradigm of glioblastoma: an analysis of global cost estimates and their utility for cost assessment. J Med Econ. 2021;24:1018-1024. 34353213 10.1080/13696998.2021.1964775

[4] Li Y , Jönsson L. The health and economic burden of brain disorders: consequences for investment in diagnosis, treatment, prevention and R&D. Cereb Circ Cogn Behav. 2025;8:100377. 39897094 10.1016/j.cccb.2025.100377 PMC11786689

[5] Bray F , Laversanne M, Sung H, et al Global cancer statistics 2022: GLOBOCAN estimates of incidence and mortality worldwide for 36 cancers in 185 countries. CA Cancer J Clin. 2024;74:229-263. 38572751 10.3322/caac.21834

[6] Chen S , Cao Z, Prettner K, et al Estimates and projections of the global economic cost of 29 cancers in 204 countries and territories from 2020 to 2050. JAMA Oncol. 2023;9:465-472. 36821107 10.1001/jamaoncol.2022.7826 PMC9951101

[7] Raggi A , Leonardi M. Burden and cost of neurological diseases: a European North–South comparison. Acta Neurol Scand. 2015;132:16-22. 25345990 10.1111/ane.12339

[8] Olesen J , Gustavsson A, Svensson M, et al; European Brain Council. The economic cost of brain disorders in Europe. Eur J Neurol. 2012;19:155-162. 22175760 10.1111/j.1468-1331.2011.03590.x

[9] Gustavsson A , Svensson M, Jacobi F, et al; CDBE2010Study Group. Cost of disorders of the brain in Europe 2010. Eur Neuropsychopharmacol. 2011;21:718-779. 21924589 10.1016/j.euroneuro.2011.08.008

[10] Miller KD , Ostrom QT, Kruchko C, et al Brain and other central nervous system tumor statistics, 2021. CA Cancer J Clin. 2021;71:381-406. 34427324 10.3322/caac.21693

[11] Chen Z , Tian F, Zhang Y. Cost-effectiveness analysis of bevacizumab combined with lomustine in the treatment of progressive glioblastoma using a Markov model simulation analysis. Front Public Health. 2024;12:1410355. 38883194 10.3389/fpubh.2024.1410355 PMC11177686

[12] Garside R , Pitt M, Anderson R, et al The effectiveness and cost-effectiveness of carmustine implants and temozolomide for the treatment of newly diagnosed high-grade glioma: a systematic review and economic evaluation. Health Technol Assess (Winchester, England). 2007;11:iii-iiv, ix. 10.3310/hta11450 17999840

[13] Price M , Ballard C, Benedetti J, et al CBTRUS statistical report: primary brain and other central nervous system tumors diagnosed in the United States in 2017–2021. Neuro-oncology. 2024;26:vi1–vi85. 39371035 10.1093/neuonc/noae145 PMC11456825

[14] Liu R , Page M, Solheim K, Fox S, Chang SM. Quality of life in adults with brain tumors: current knowledge and future directions. Neuro Oncol. 2009;11:330-339. 19001097 10.1215/15228517-2008-093 PMC2718978

[15] Manzano A , Svedman C, Hofmarcher T, Wilking N. Comparator Report on Cancer in Europe 2025–Disease Burden, Costs and Access to Medicines and Molecular Diagnostics. Lund, Sweden: IHE-report-2025_2_ The Swedish Institute for Health Economics; 2025.

[16] Pöhlmann J , Weller M, Marcellusi A, et al High costs, low quality of life, reduced survival, and room for improving treatment: an analysis of burden and unmet needs in glioma. Front Oncol. 2024;14:1368606. 38571509 10.3389/fonc.2024.1368606 PMC10987841

[17] Institute of Clinical and Economic Review. Cost-Effectiveness, the QALY, and the evLY. ICER; 2024. https://icer.org/our-approach/methods-process/cost-effectiveness-the-qaly-and-the-evlyg/. Accessed September 6, 2025.

[18] Butenschoen VM , Kelm A, Meyer B, Krieg SM. Quality-adjusted life years in glioma patients: a systematic review on currently available data and the lack of evidence-based utilities. J Neurooncol. 2019;144:1-9. 31187319 10.1007/s11060-019-03210-2

[19] Gupta S , Smith TR, Broekman ML. Ethical considerations of neuro-oncology trial design in the era of precision medicine. J Neurooncol. 2017;134:1-7. 28555425 10.1007/s11060-017-2502-0 PMC5543186

[20] Wouterse B , van Baal P, Versteegh M, Brouwer W. The value of health in a cost-effectiveness analysis: theory versus practice. Pharmacoeconomics. 2023;41:607-617. 37072598 10.1007/s40273-023-01265-8 PMC10163089

[21] World Health Organizantion. Global Spending on Health: Emerging from the Pandemic. World Health Organization; 2024.

[22] Martin AB , Hartman M, Washington B, Catlin A; Team NHEA. National health expenditures in 2023: faster growth as insurance coverage and utilization increased: article examines national health expenditures in 2023. Health Aff (Millwood). 2025;44:12-22. 39693578 10.1377/hlthaff.2024.01375

[23] Thomas R , Chalkidou K. Cost-Effectiveness Analysis. Health System Efficiency; 2016.

[24] Schaff LR , Mellinghoff IK. Glioblastoma and other primary brain malignancies in adults: a review. JAMA. 2023;329:574-587. 36809318 10.1001/jama.2023.0023 PMC11445779

[25] Holdhoff M , Grossman SA. Controversies in the adjuvant therapy of high-grade gliomas. Oncologist. 2011;16:351-358. 21339260 10.1634/theoncologist.2010-0335 PMC3228107

[26] PRISMA. PRISMA 2020; 2020. https://www.prisma-statement.org/prisma-2020. Accessed April 5, 2025.

[27] Husereau D , Drummond M, Augustovski F, et al Consolidated Health Economic Evaluation Reporting Standards 2022 (CHEERS 2022) statement: updated reporting guidance for health economic evaluations. MDM Policy Pract. 2022;7:23814683211061097. 35036563 10.1177/23814683211061097 PMC8755935

[28] Group WB. GDP per capita (current US$). 2024. https://data.worldbank.org/indicator/NY.GDP.PCAP.CD. Accessed September 19, 2025.

[29] Shemilt I. CCEMG-EPPI-Centre Cost Converter; Version 1.4. The Campbell and Cochrane Economics Methods Group (CCEMG) and the Evidence for Policy and Practice Information and Coordinating Centre (EPPI-Centre); 2014.

[30] Shemilt I , James T, Marcello M. A web-based tool for adjusting costs to a specific target currency and price year. Evid Policy. 2010;6:51-59.

[31] Manuel SP , Chia ZK, Raygor KP, Fernández A. Association of language barriers with process outcomes after craniotomy for brain tumor. Neurosurgery. 2022;91:590-595. 35857019 10.1227/neu.0000000000002080 PMC10552977

[32] Martino J , Gomez E, Bilbao JL, Dueñas JC, Vázquez-Barquero A. Cost-utility of maximal safe resection of WHO grade II gliomas within eloquent areas. Acta Neurochir (Wien). 2013;155:41-50. 23132374 10.1007/s00701-012-1541-8

[33] Nassiri F , Li L, Badhiwala JH, et al Hospital costs associated with inpatient versus outpatient awake craniotomy for resection of brain tumors. J Clin Neurosci. 2019;59:162-166. 30414812 10.1016/j.jocn.2018.10.110

[34] Neville IS , Ureña FM, Quadros DG, et al Safety and costs analysis of early hospital discharge after brain tumour surgery: a pilot study. BMC Surg. 2020;20:105. 32410602 10.1186/s12893-020-00767-y PMC7227314

[35] Tang OY , Perla KMR, Lim RK, Yoon JS, Weil RJ, Toms SA. Interhospital competition and hospital charges and costs for patients undergoing cranial neurosurgery. J Neurosurg. 2021;135:361-372. 33007751 10.3171/2020.6.JNS20732

[36] Ter Laan M , Roelofs S, Van Huet I, Adang EM, Bartels RH. Selective intensive care unit admission after adult supratentorial tumor craniotomy: complications, length of stay, and costs. Neurosurgery. 2020;86:E54-E59. 31541243 10.1093/neuros/nyz388 PMC6911731

[37] Ter Laan M , Roelofs S, Adang EM, Bartels RH. Reducing the burden of brain tumor surgery. Acta Neurochir (Wien). 2021;163:1879-1882. 32870422 10.1007/s00701-020-04543-y PMC8195912

[38] Zhang Y , Zhang M, Lin M, et al Costs and complications associated with resection of supratentorial tumors with and without the operative microscope in the United States. World Neurosurg. 2020; 138: e607–e19. 32171932 10.1016/j.wneu.2020.03.021 PMC9288831

[39] Zygourakis CC , Liu CY, Yoon S, et al Analysis of cost variation in craniotomy for tumor using 2 national databases. Neurosurgery. 2017;81:972-979. 28402457 10.1093/neuros/nyx133

[40] Growth Lab. The atlas of economic complexity; 2024. https://atlas.hks.harvard.edu/. Accessed September 19, 2025.

[41] Banegas MP , Hassett MJ, Keast EM, et al Patterns of medical care cost by service type for patients with recurrent and de novo advanced cancer. Value Health. 2022;25:69-76. 35031101 10.1016/j.jval.2021.06.016

[42] Phillips KR , Enriquez-Marulanda A, Mackel C, et al Predictors of extended length of stay related to craniotomy for tumor resection. World Neurosurg X. 2023;19:100176. 37123627 10.1016/j.wnsx.2023.100176 PMC10139985

[43] Dasenbrock HH , Liu KX, Devine CA, et al Length of hospital stay after craniotomy for tumor: a national surgical quality improvement program analysis. Neurosurg Focus. 2015;39:E12. 10.3171/2015.10.FOCUS15386 26621410

[44] Hoffman SE , Gupta S, O’Connor M, et al Reduced time to imaging, length of stay, and hospital charges following implementation of a novel postoperative pathway for craniotomy. J Neurosurg. 2023;139:373-384. 36609368 10.3171/2022.12.JNS222123 PMC10904334

[45] Missios S , Bekelis K. Drivers of hospitalization cost after craniotomy for tumor resection: creation and validation of a predictive model. BMC Health Serv Res. 2015;15:85. 25756732 10.1186/s12913-015-0742-2 PMC4351828

[46] Jackson HN , Hadley CC, Khan AB, et al Racial and socioeconomic disparities in patients with meningioma: a retrospective cohort study. Neurosurgery. 2022;90:114-123. 34982878 10.1227/NEU.0000000000001751 PMC9514723

[47] Spadola M , Farooqi A, Dimentberg R, et al The effect of household income on outcomes following supratentorial meningioma resection. Clin Neurol Neurosurg. 2020;195:106031. 32652393 10.1016/j.clineuro.2020.106031

[48] Chan AY , Choi EH, Oh MY, et al Elective versus nonelective brain tumor resections: a 5-year propensity score matching cost comparison analysis. J Neurosurg. 2022;136:40-44. 34243148 10.3171/2020.12.JNS203401

[49] Sun R , Sharma S, Benghiat H, et al Reconfiguration from emergency to urgent elective neurosurgery for glioblastoma patients improves length of stay, surgical adjunct use, and extent of resective surgery. Neurooncol Pract. 2022;9:420-428. 36127892 10.1093/nop/npac034 PMC9476969

[50] Chan P-C , Rodrigues MM, Lim FM-Y, Chow E, Lo S-H. Economic burden of brain metastases in patients with non-small cell lung cancer: costs and implications. Ann Palliat Med. 2019;8:210-214. 30691277 10.21037/apm.2018.12.03

[51] Liu Y , Tyler E, Lustick M, Klein D, Walter KA. Healthcare costs for high-grade glioma. Anticancer Res. 2019;39:1375-1381. 30842171 10.21873/anticanres.13251

[52] Crooks J , Dominic O, Shepard M, et al Cost of treatment for brain metastases using data from a national health insurance provider. Adv Radiat Oncol. 2024;9:101438. 38567144 10.1016/j.adro.2024.101438 PMC10985802

[53] Ray S , Dacosta-Byfield S, Ganguli A, Bonthapally V, Teitelbaum A. Comparative analysis of survival, treatment, cost and resource use among patients newly diagnosed with brain metastasis by initial primary cancer. J Neurooncol. 2013;114:117-125. 23700325 10.1007/s11060-013-1160-0

[54] Wager E , McGough M, Rakshit S, Cox C. How Does Health Spending in the U.S. compare to Other Countries? Peterson-KFF Health System Tracker; 2025. https://www.healthsystemtracker.org/chart-collection/health-spending-u-s-compare-countries/. Accessed September 21, 2025.

[55] Health Care Cost Institute. 2022 Health Care Cost And Utilitzation Report. Health Care Cost Institute; 2024.

[56] Senders JT , Maas SLN, Draaisma K, et al International practice variation in perioperative laboratory testing in glioblastoma patients—a retrospective cohort study. Acta Neurochir (Wien). 2022;164:385-392. 34997355 10.1007/s00701-021-05090-w PMC8854260

[57] Finkler SA. The distinction between cost and charges. Ann Intern Med. 1982;96:102-109. 7053682 10.7326/0003-4819-96-1-102

[58] Tebha SS , Memon SA, Mehmood Q, Mukherjee D, Abdi H, Negida A. Glioblastoma management in low and middle-income countries; existing challenges and policy recommendations. Brain Spine. 2023;3:101775. 38021027 10.1016/j.bas.2023.101775 PMC10668069

[59] Paiva ALC , Vitorino-Araujo JL, Lovato RM, da Costa GHF, Veiga JCE. An economic study of neuro-oncological patients in a large developing country: a cost analysis. Arq Neuropsiquiatr. 2022;80:1149-1158. 36577414 10.1055/s-0042-1758649 PMC9797276

[60] Koopmanschap MA , Rutten FF. Indirect costs in economic studies: ­confronting the confusion. Pharmacoeconomics. 1993;4:446-454. 10146911 10.2165/00019053-199304060-00006

[61] Tunthanathip T , Sae-Heng S, Oearsakul T, et al Quality of life, out-of-pocket expenditures, and indirect costs among patients with the central nervous system tumors in Thailand. J Neurosci Rural Pract. 2022;13:740-749. 36743773 10.25259/JNRP-2022-3-45 PMC9894017

[62] Undabeitia J , Torres-Bayona S, Samprón N, et al Costes indirectos asociados al glioblastoma. Experiencia en un centro. Neurología. 2018;33:85-91. 27449154 10.1016/j.nrl.2016.05.003

[63] Willis MS , Nilsson A, Neslusan CA. A review of heterogeneity in comparative economic analysis, with specific considerations for the decentralized US setting and patient-centered care. Pharmacoeconomics. 2025;43:601-616. 40057662 10.1007/s40273-025-01478-z PMC12081492

[64] Au K , Bharadwaj S, Venkatraghavan L, Bernstein M. Outpatient brain tumor craniotomy under general anesthesia. J Neurosurg. 2016;125:1130-1135. 26943840 10.3171/2015.11.JNS152151

[65] Bernstein M. Outpatient craniotomy for brain tumor: a pilot feasibility study in 46 patients. Can J Neurol Sci. 2001;28:120-124. 11383935 10.1017/s0317167100052781

[66] Boulton M , Bernstein M. Outpatient brain tumor surgery: innovation in surgical neurooncology. J Neurosurg. 2008;108:649-654. 18377241 10.3171/JNS/2008/108/4/0649

[67] Vallejo FA , Eichberg DG, Morell AA, et al Same-day discharge after brain tumor resection: a prospective pilot study. J Neurooncol. 2022;157:345-353. 35192136 10.1007/s11060-022-03969-x PMC8861287

[68] Peláez-Sanchez CA , Pajarón-Guerrero M, Rodriguez-Caballero A, et al Cost analysis of oncological outpatient neurosurgery under general anesthesia with hospital-at-home-based postoperative care. World Neurosurg. 2025;193:1002-1007. 39481839 10.1016/j.wneu.2024.10.093

